# Bioinformatics analysis of hub genes as osteoarthritis prognostic biomarkers

**DOI:** 10.1038/s41598-023-48446-1

**Published:** 2023-12-21

**Authors:** Junfeng Zeng, Xinhao Jiang, Mo Jiang, Yuexia Cao, Yi Jiang

**Affiliations:** 1 Department of Orthopedics, The First Affiliated Hospital, Jiangxi Medical College, Nanchang University, No. 17, Yongwaizheng Street, Donghu District, Nanchang City, Jiangxi Province 330000 People’s Republic of China; 2 Department of Orthopedics, Yugan County Hospital, No. 1, Mianshan Avenue, Yugan County, Shangrao City, Jiangxi Province 335100 People’s Republic of China; 3 Department of Orthopedics 10th, The Affiliated Hospital of Jiangxi University of Traditional Chinese Medicine, No. 445, Bayi Avenue, Donghu District, Nanchang City, Jiangxi Province 330000 People’s Republic of China

**Keywords:** Biological techniques, Biotechnology, Cell biology, Genetics

## Abstract

Osteoarthritis (OA) is a progressive cartilage degradation disease, concomitant with synovitis, osteophyte formation, and subchondral bone sclerosis. Over 37% of the elderly population is affected by OA, and the number of cases is increasing as the global population ages. Therefore, the objective of this study was to identify and analyze the hub genes of OA combining with comprehensive bioinformatics analysis tools to provide theoretical basis in further OA effective therapies. Two sample sets of GSE46750 contained 12 pairs OA synovial membrane and normal samples harvested from patients as well as GSE98918 including 12 OA and non-OA patients were downloaded from the Gene Expression Omnibus database (GEO) database. Differentially expressed genes (DEGs) were identified using Gene Expression Omnibus 2R (GEO2R), followed by functional enrichment analysis, protein–protein interaction networks construction. The hub genes were identified and evaluated. An OA rat model was constructed, hematoxylin and eosin staining, safranin O/fast green staining, cytokines concentrations of serum were used to verify the model. The hub genes expression level in the knee OA samples were verified using RT-qPCR. The top 20 significantly up-regulated and down-regulated DEGs were screened out from the two datasets, respectively. The top 18 GO terms and 10 KEGG pathways were enriched. Eight hub genes were identified, namely *MS4A6A*, *C1QB*, *C1QC*, *CD74*, *CSF1R*, *HLA-DPA1*, *HLA-DRA* and *ITGB2*. Among them, the hub genes were all up-regulated in in vivo OA rat model, compared with healthy controls. The eight hub genes identified (*MS4A6A*, *C1QB*, *C1QC*, *CD74*, *CSF1R*, *HLA-DPA1*, *HLA-DRA* and *ITGB2*) were shown to be associated with OA. These genes can serve as disease markers to discriminate OA patients from healthy controls.

## Introduction

Osteoarthritis (OA) is characterised by progressive cartilage degradation, synovitis, osteophyte formation, and subchondral bone sclerosis^1^. Knee OA is one of the most common types of OA, affecting 37% of persons aged 60 years or older^2^. The prevalence of OA is expected to increase due to global aging^3^. In many previous studies of OA, synovitis plays no role or only severe forms of synovitis increase risk in OA, which resulted in a serious lack of understanding of the inflammatory conditions of the OA synovial fluids^4–6^. In fact, synovitis has been shown to be an independent risk factor for OA^7^ and is one of the important pathological factors of the vicious cycle perpetuating OA^8^. Compared with healthy synovial fluids, OA synovial fluids are known to be rich in inflammatory mediators, such as chemokines, cytokines, and complement components^9^, including tumor necrosis factor α (TNF-α), Interleukin-1β (IL-1β), Interleukin-6 (IL-6), matrix metalloproteinase-1 and matrix metalloproteinase-13, as well as nitric oxide (NO), prostaglandin E2, granulocyte macrophage colony-stimulating factor (GM-CSF), and vascular cell adhesion molecule-1^9^. Synovitis causes articular cartilage and meniscus degeneration^10^, and the expression and activation of complement components, in particular, contributes to the progression of chondropathy^11^. The alternative pathway of complement seems to play a crucial role in the pathogenesis of OA. The C3 and its activating peptide C3a have been shown to belong to the alternative pathway of complement, as they have been detected at high levels in OA cartilage, synovitis membrane tissues, and cultured chondrocytes^12^.

There are currently no effective methods to screen for OA at the early, asymptomatic stage of the disease. New disease markers are urgently needed in order to identify OA in patients before disease progression. Bioinformatics-based methods offer great advantages in the discovery of new disease markers. Xia et al. conducted an in-depth bioinformatics analysis of OA synovitis samples and found seven unreported hub genes in the ferroptosis signalling pathway that could be used as OA-associated markers^13^. Liu et al*.* reported SLC3A2 to be a ferroptosis signalling pathway protein with clinical value and as a potential therapeutic target in treatment of OA, based on bioinformatics analysis^14^. In another bioinformatics study, Wang et al*.* discovered four cuproptosis-related hub genes that were closely related to OA inflammatory microenvironments and had clinical application values as markers of OA^15^.

The objective of this study was to identify potential hub genes, biomarkers and molecular processes involved in OA progression combining comprehensive bioinformatics analysis, which further verified in vivo OA rat model. The study may contribute to providing new references for identification of the molecular therapeutic targets in further OA effective therapies.

## Materials and methods

### Datasets

Two raw datasets of GSE46750 and GSE98918 were downloaded from the Gene Expression Omnibus database (GEO) (https://www.ncbi.nlm.nih.gov/geo/). GSE46750 contained 12 pairs OA synovial membrane (10 women and 2 men; mean age 70 years, range 50–83 years) and normal samples harvested from patients (10 women and 2 men; mean age 70 years, range 50–83 years). GSE98918 contained 12 OA (9 women and 3 men; mean age 65 years; mean body-mass-index: 36) and non-OA patients (5 women and 7 men; mean age 49 years; mean body-mass-index: 27).

### Identification of differentially expressed genes (DEGs)

The DEGs in the two datasets were identified by searching the Gene Expression Omnibus 2R (GEO2R) (https://www.ncbi.nlm.nih.gov/geo/geo2r) that met the criteria of *p* ≤ 0.05 and |log2FC| ≥ 1 (OA vs. control). The DEGs were finally determined using a Benjamini–Hochberg FDR (false discovery rate) multiple testing correction, and *p* value was analyzed to correct false-positive results, which *p* ≤ 0.05 and |log2FC| ≥ 1 was defined as the threshold. Heat maps and volcano maps were drawn to visualize the DEGs. The two datasets were normalised and cross-comparable evaluation was visualised using boxplots.

### Gene ontology (GO) terms and Kyoto Encyclopedia of Genes and Genomes (KEGG) pathway enrichment analysis

Database for Annotation, Visualization and Integrated Discovery (DAVID): Functional Annotation Tool (https://david.ncifcrf.gov/summary.jsp) was used to perform GO term and KEGG pathway enrichment analysis of common DEGs (co-DEGs). R language package was used to perform and visualize the enrichment analysis results with the thresholds of *p* value < 0.05 and enrichment gene count ≥ 2. The bar graph and bubble plot were drawn.

### Construction of protein–protein interaction (PPI) network and identification of hub genes

The Search Tool for the Retrieval of Interacting Genes/Proteins (STRING) online database (https://www.string-db.org/) was used to construct the PPI network of DEGs that may play important roles in the progression of OA. The confidence interaction score was set at 0.15 as significance criteria. PPI network was performed using Cytoscape (www.cytoscape.org/) to better visualize the interaction information. Key modules were identified using MCODE (the Molecular Complex Detection) following filter criteria: degree cut-off = 2; node score cut-off = 0.2; k-core = 2; and max depth = 100. The hub genes were selected out with the connectivity degree ≥ 10 using CytoHubba Version 0.1 plugin.

### Verification of hub genes

The hub genes GSEA ridgeplot was drawn using R language package. The receiver operating characteristic (ROC) curves of the hub genes were drawn using the Gene Expression Profiling Interactive Analysis (GEPIA) (http://gepia.cancer-pku.cn/). The area under curve (AUC) of the corresponding ROC curves of the hub genes were used to evaluate the discriminative effects of OA tissues from healthy controls. The expression profiles of the hub genes in the two datasets were used as variates to perform the principal component analysis (PCA), and PC1 and PC2 were obtained.

### Construction of OA rat model

All the animal experiments were approved by the Animal Care and Use Committee of our hospital and were conducted in accordance with the NIH Guidelines for the Care and Use of Laboratory Animals. Twelve 6–8 weeks old healthy male Sprague–Dawley rats (180–200 g of body weight) were purchased from SPF (Beijing) Biotechnology Co., Ltd. (Beijing, China). The rats were housed in a standard specific pathogen-free animal centre that provided the condition of controlled temperature and humidity and 12 h/12 h light–dark cycles. Before the OA-inducing operation was conducted, the rats were adaptively fed for 1 week.

Rats were randomly divided into two groups, the control group and OA group (n = 6). Knee OA was induced by intra-articular injection of monosodium iodoacetate (MIA) (Cat. No. 57858, Sigma-Aldrich) as described previously^16^. Rats were anesthetised by intraperitoneal injection [i.p.] of a pentobarbital sodium (50 mg/kg) and ketamine (25 mg/kg) mixture. The rat’s right hind limb was fixed so that it could arch straight up and protrude the joint cavity; the other limbs were left alone. On the first day, OA group rats were given intra-articular injection of 2 mg MIA (in 40 μL normal saline) to induce osteoarthritis of the knee. Control group mice were given 40 μL normal saline only. After 4 weeks of induction, all rats were sacrificed by CO_2_ asphyxiation. The bilateral knee joints and tissues were harvested and then immediately frozen in liquid nitrogen and stored in a − 80 °C refrigerator for later use.

### Histology analysis

Haematoxylin and eosin or safranin O/fast green stained sections were utilized to evaluate matrix proteoglycan and overall joint morphology. The rat knee joints were fixed with 4% paraformaldehyde at 4 °C for more than 48 h and embedded in paraffin. Tissue sections of 5 μm thickness were dewaxed using 100% xylene and hydrated using gradient alcohol (70–80–90–95–100% alcohol). The prepared tissue sections were then stained with haematoxylin and eosin or safranin O/fast green for 10 min at room temperature, followed by application of hydrochloric acid ethanol solution for 3 s, 95% alcohol for 30 s, and gradient alcohol (95–95–100–100%) for 30 s. Lastly, the tissue sections were treated with xylene twice, 30 s each time, to each transparency, after which the sections were placed in a neutral mounting medium (Cat. No. KGF0281, KeyGEN Bio Tech, Jiangsu, China) and observed under microscope (ECLIPSE Ni-E, Nikon, Japan).

### ELISA

Before the rats were sacrificed, blood samples were collected from the orbital venous plexus. Then blood samples were stored at 4 °C for 2 h and incubated in 65 °C water bath for 15 min. The serum samples were collected from the top layer and cooled at 4 °C. The levels of serum TNF-α, IL-1β and IL-6 levels were then determined using Enzyme-linked immunosorbent assay (ELISA) according to the manufactures’ instructions. The kits used were as follows: serum TNF-α ELISA kit (Cat. No. CSB-E11987r, CUSABIO, Wuhan, China), serum IL-1β ELISA kit (Cat. No. CSB-E08055r, CUSABIO, Wuhan, China), and serum IL-6 ELISA kit (Cat. No. CSB-E04640r, CUSABIO, Wuhan, China).

### Quantitative real time PCR (RT-qPCR)

Rats knee joints tissues were homogenised and lysed with TRIzol reagent (Cat. No. 15596026, Thermo Fisher) in liquid nitrogen. Total RNA was extracted using total RNA extraction kit (Cat. No. RC101-01, Vazyme, Nanjing, China). Reverse transcription synthesis of cDNA was conducted using cDNA first strand reverse transcription synthesis kit (Cat. No. 6210A, TaKaRa, Beijing, China). RT-qPCR was performed using TB Green Fast qPCR Mix (Cat. No. RR001B, TaKaRa, Beijing, China). Relative gene expression was calculated using the 2^−ΔΔCt^ method with GAPDH used for normalization of the results. The primer sequences are provided in Supplementary Table 1.

### Statistics

All data processing and analysis were performed in Grand prism 7.0. To compare two groups of variables, an independent Student’s t-test was applied. Fisher’s exact test was carried out to analyse the statistical significance between two datasets of variables. *p* < 0.05 was considered statistically significant.

### Ethical approval

All the animal experiments were approved by the Animal Care and Use Committee of the First Affiliated Hospital, Jiangxi Medical College, Nanchang University and were conducted in accordance with the NIH Guidelines for the Care and Use of Laboratory Animals. The study is reported in accordance with ARRIVE guidelines.

## Results

### Identification of DEGs

**Figure 1:**
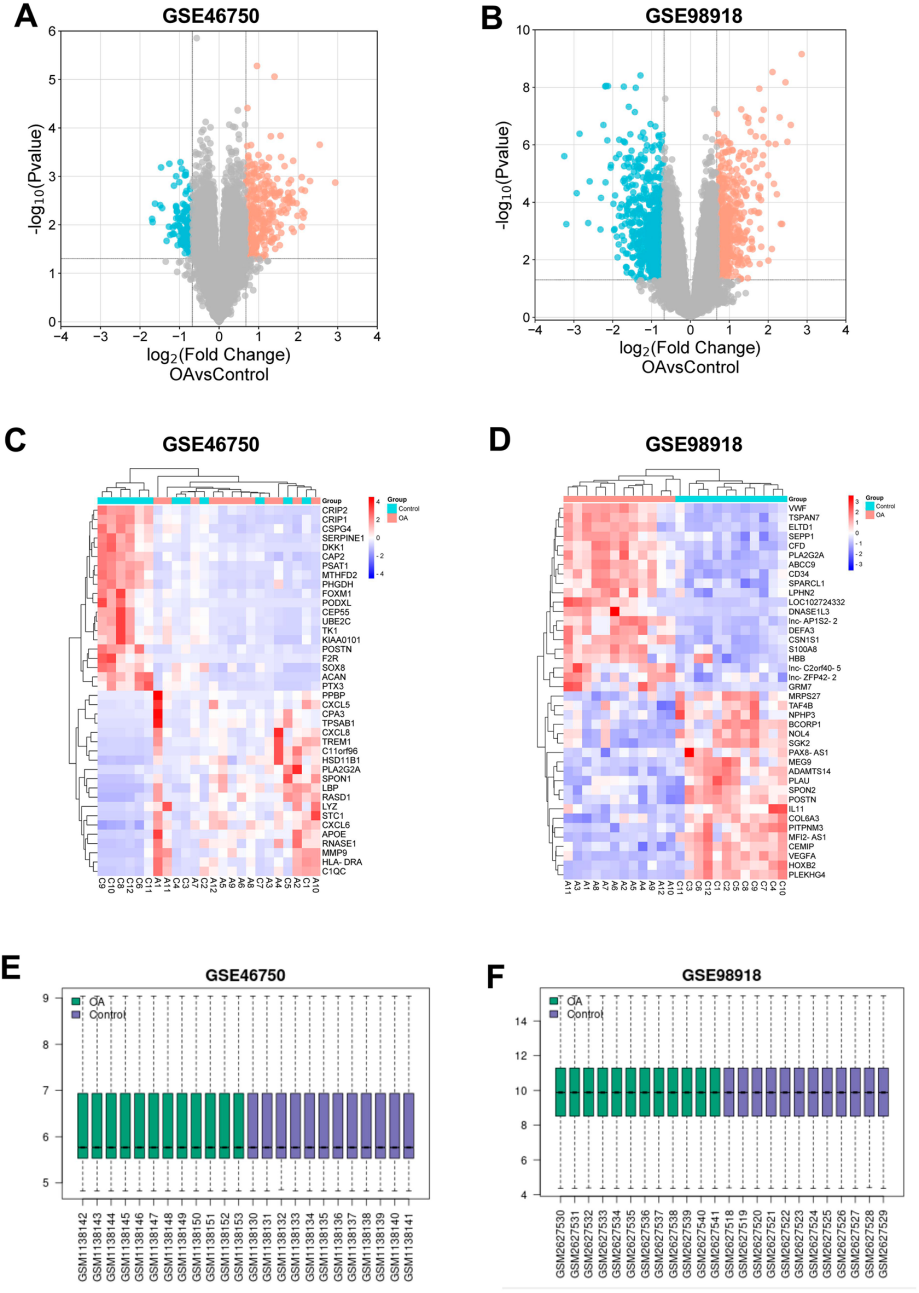
The volcano plots and heat maps of GSE46750 and GSE98918 datasets. (**A**) The volcano plot of GSE46750 dataset. The x-axis represents log2 (Fold Change) and the y-axis represents − log10 (*p* value). Red dots indicate up-regulated genes and blue dots indicate down-regulated genes. (**B**) The volcano plot of GSE98918 dataset. (**C**) The heat map of GSE46750 dataset. Every line represents one gene and every column represents one sample. Red colour indicates high-expression level and blue colour indicates low-expression level. (**D**) The heat map of GSE98918 dataset. (**E**) The cross-comparability evaluation of GSE46750 dataset. (**F**) The cross-comparability evaluation of GSE98918 dataset.

**Figure 2:**
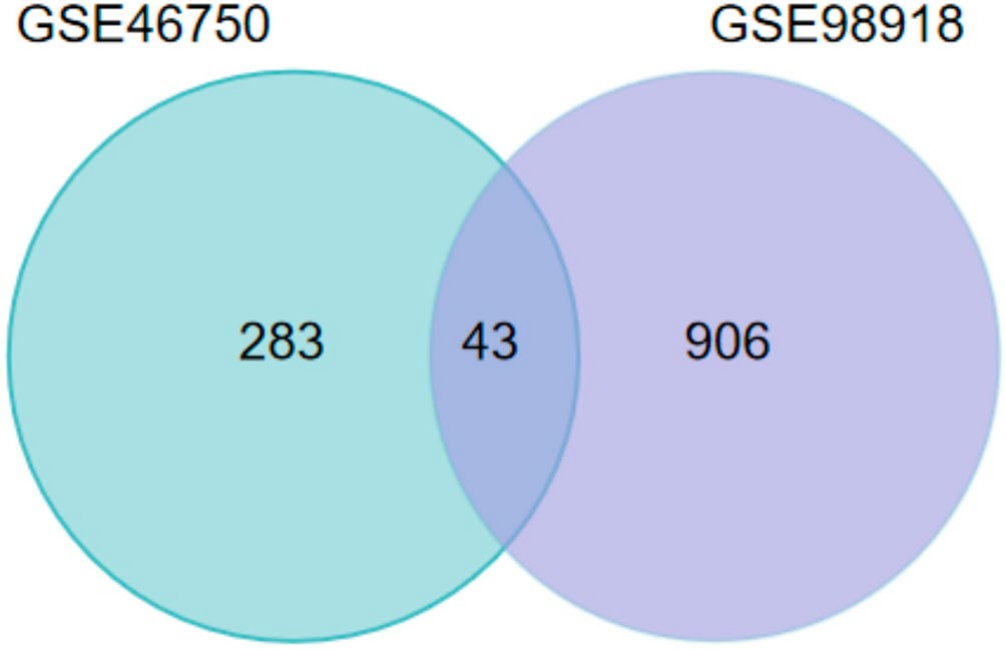
Venn diagram of common DEGs in both GSE46750 and GSE98918 datasets.

Based on the bioinformatic analysis through GEO2R with the criteria of *p* ≤ 0.05 and |log2FC| ≥ 1, 326 DEGs of GSE46750 dataset and 949 DRGs of GSE98918 dataset were identified. Subsequently, cluster analysis of these DEGs was performed. The top 20 up-regulated and down-regulated DEGs of GSE46750 and GSE98918 datasets were prioritized, respectively. Details are shown in Supplementary Tables 2–3. The volcano map and heat map were drawn based on the cluster analysis of GSE46750 (Fig. 1A,C) and GSE98918 (Fig. 1B,D). As can be seen in the heat maps, the sample clustering showed high confidence. After normalization and cross-comparable evaluation (GSE46750 dataset normalization is shown in Fig. 1E, and that of GSE98918 in Fig. 1F), it was shown that the data distribution of the two sample sets met the standard criteria, indicating that the microarray data was of high quality and cross-comparability. Venn diagram was drawn to show the common DEGs of the two datasets (Fig. 2); there were 43 co-DEGs shared between the two datasets.

### Enrichment analysis of DEGs

**Figure 3:**
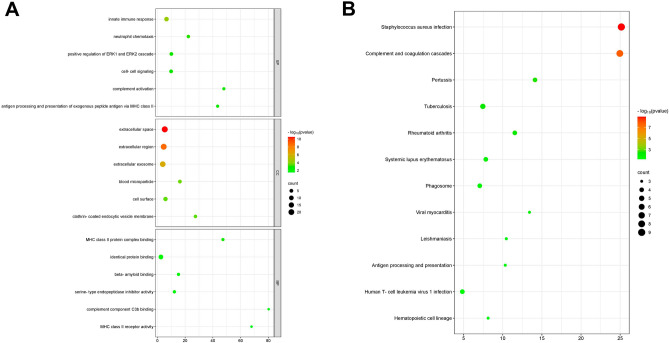
Bubble plots of GO and KEGG pathway enrichment analysis results. (**A**) Bubble plots visualized the Gene Ontology (GO) enrichment analysis results of common DEGs (co-DEGs). The different depths of nodes’ color represent the different adjusted *p* value. The different sizes of the nodes represent the different number of genes. (**B**) Bubble plots visualized the Kyoto Encyclopedia of Genes and Genome (KEGG) pathway enrichment analysis results of common DEGs (co-DEGs).

GO and KEGG pathway enrichment analyses were performed to evaluate the function of the 43 co-DEGs (Fig. 3). The categories of GO terms include BP (biological process), CC (cellular component), and MF (molecular function). The top enriched 6 GO terms in each category met the criteria with the lowest *p* value were selected (Supplementary Table 4) and visualised using bubble plots (Fig. 3A). The bar graph of GO enrichment analysis was shown in Supplement Fig. 1A. The co-DEGs were mainly enriched in the GO terms of “complement activation”, “clathrin-coated endocytic vesicle membrane”, “complement component C3b binding”. The top 10 pathways with the lowest *p* value were enriched using KEGG pathway enrichment analysis (Supplementary Table 5) and visualised using bubble plots (Fig. 3B). The bar graph of KEGG pathway enrichment analysis was shown in Supplement Fig. 1B. The co-DEGs were mainly enriched in the KEGG pathways of “Staphylococcus aureus infection”, “Complement and coagulation”, “Pertussis”, “Tuberculosis”, “Rheumatoid arthritis”, “Systemic lupus erythematosus”, “Phagosome”, “Viral myocarditis”, “Leishmaniasis”, “Antigen processing”^17–19^.

### PPI network and hub genes

**Figure 4:**
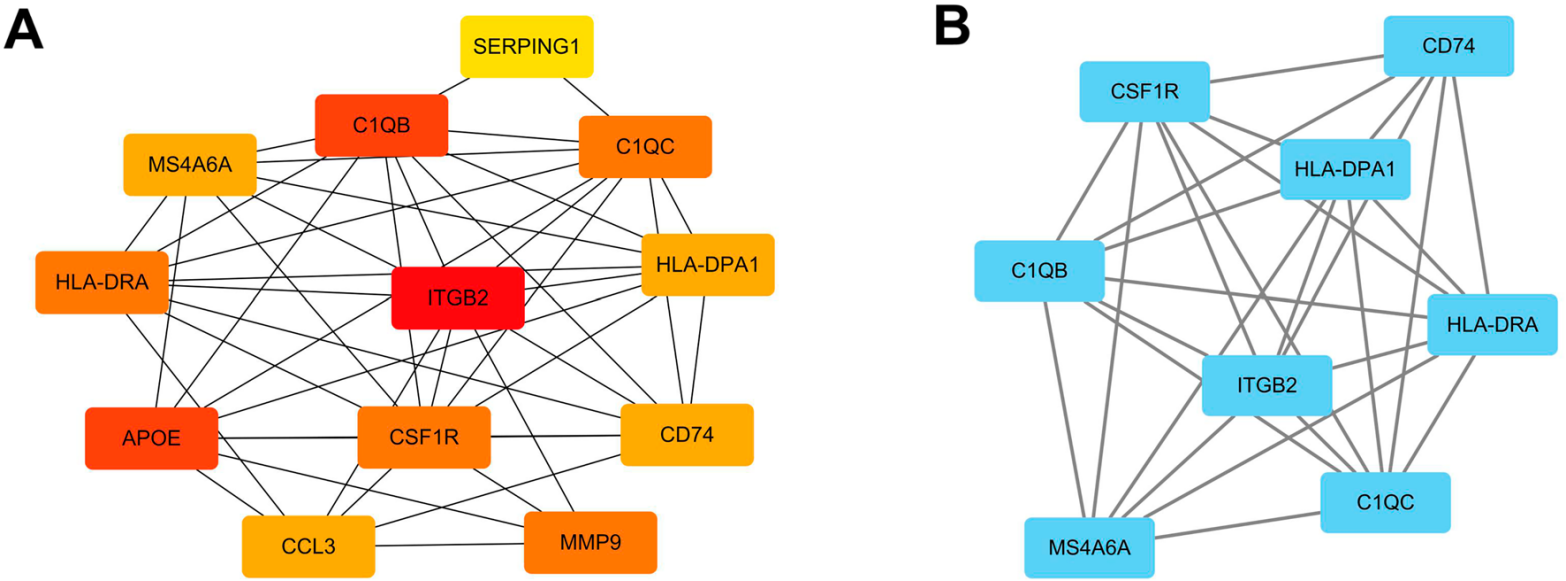
PPI network analysis and hub genes identification. (**A**) A key cluster with 12 genes was identified by MCODE. (**B**) Top 8 hub genes explored by CytoHubba.

DEGs were uploaded onto the STRING database and the PPI network was constructed (Supplement Fig. 2). PPI network visualization was performed using Cytoscape. Then a tightly connected protein cluster with 12 genes was identified through MCODE (Fig. 4A). With the degree algorithm of plug-in CytoHubba, 8 candidate hub genes were obtained, including *MS4A6A*, *C1QB*, *C1QC*, *CD74*, *CSF1R*, *HLA-DPA1*, *HLA-DRA* and *ITGB2* (Fig. 4B).

### Prognostic value of the hub genes

**Figure 5:**
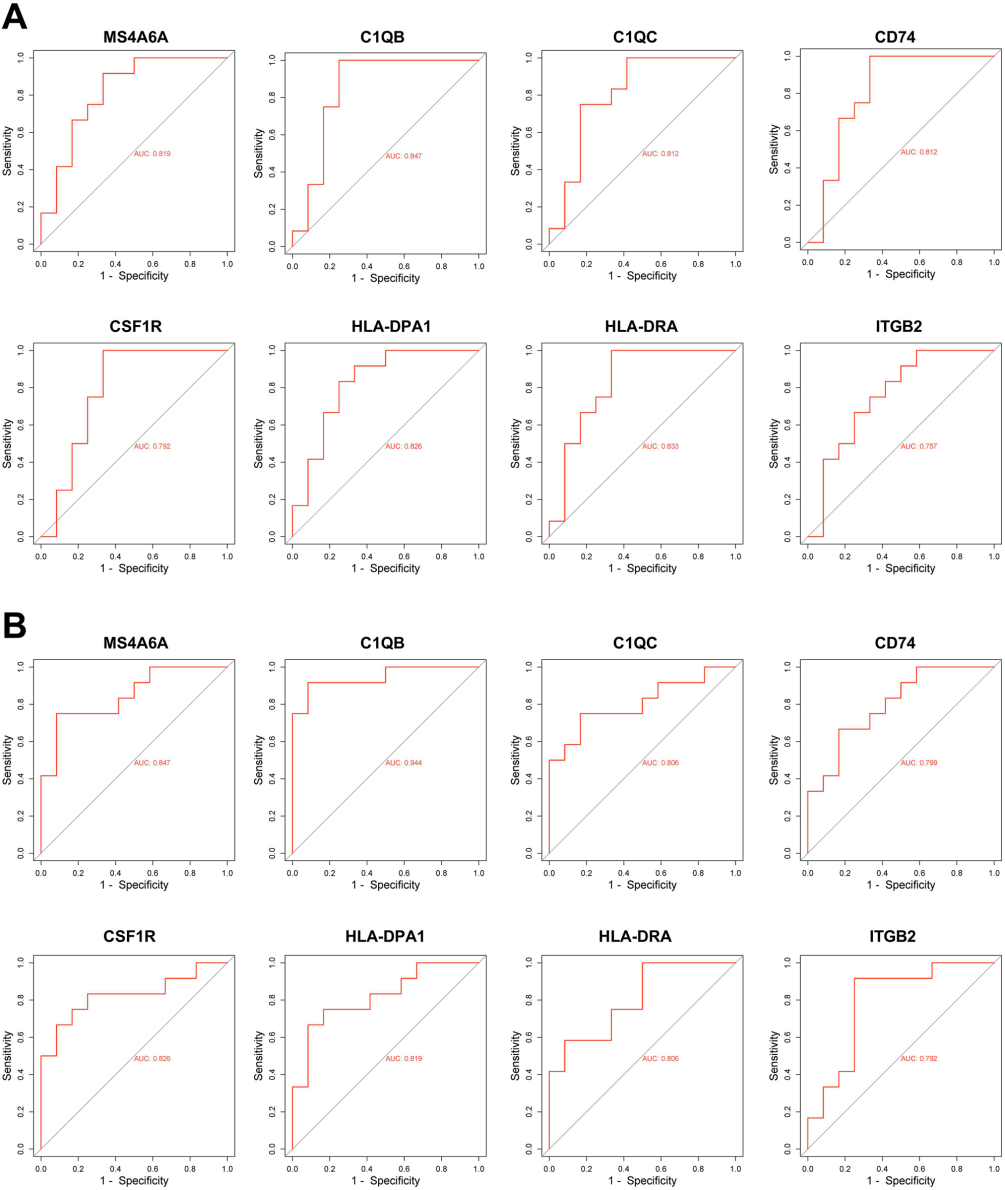
Hub genes ROC curves. (**A**) ROC curve analysis of hub genes including *MS4A6A*, *C1QB*, *C1QC*, *CD74*, *CSF1R*, *HLA-DPA1*, *HLA-DRA* and *ITGB2* in the GSE46750 dataset. (**B**) ROC curve analysis of hub genes in the GSE98918 dataset.

**Figure 6:**
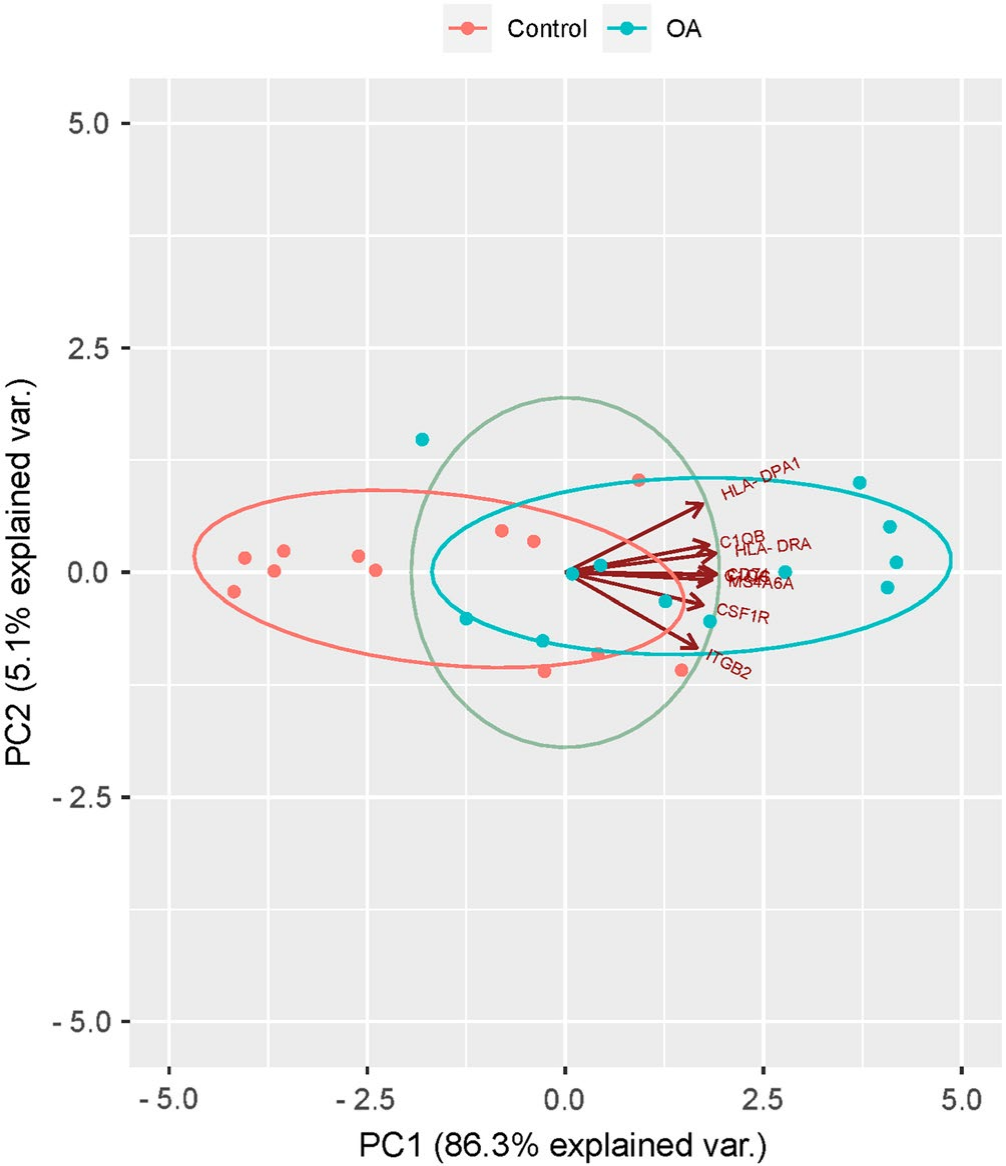
PCA plot of the six hub genes. PC1 and PC2 were the first and second principal components, which were the potential variants’ explanation effect on different expression, respectively. Plots represent different samples, and different colors represent different groups.

The ROC curves of the six hub genes were drawn based on the expression levels in both GSE46750 and GSE98918 datasets to verify the discriminative effect on synovitis tissues of OA patients from healthy controls (Fig. 5A,B). The ridgeplot of the 8 genes was drawn using R language package based on the hub genes expression in GSE46750 dataset and the distribution of each hub gene was relatively dense (Supplement Fig. 3). PCA was conducted based on the expression profiles of the hub genes in the two datasets. PC1 and PC2 variants were obtained, and combined the two of which could provide 91.4% explained variation. In the PCA plot drawn using PC1 and PC2 as x-axis and y-axis, respectively, the OA and healthy control groups samples were well separated, indicating the powerful discriminative effects of the 8 hub genes (Fig. 6).

### Verification through in vivo OA rat model

**Figure 7:**
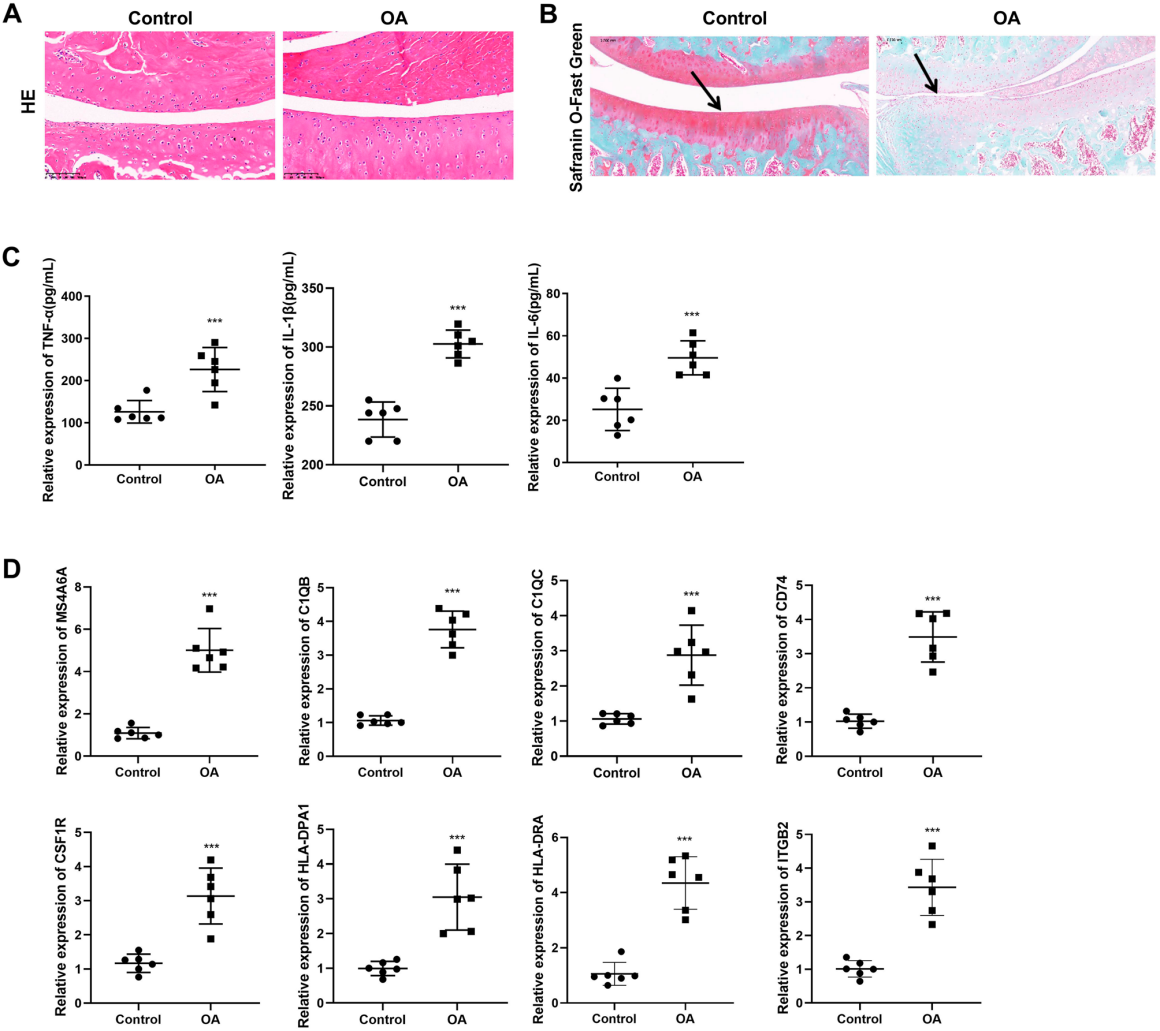
Verification hub genes’ expression in OA rat model. (**A**) HE staining of the growth plates of control and OA rat model joints. Scale bar: 100 μm. (200×) (**B**) The growth plates of control and OA rat model joints were stained using Safranin-O & fast green. Scale bar: 100 μm. (200×) (**C**) ELISA results of inflammatory cytokines levels in control and OA rat model serum. (**D**) RT-qPCR results of *MS4A6A*, *C1QB*, *C1QC*, *CD74*, *CSF1R*, *HLA-DPA1*, *HLA-DRA* and *ITGB2* in control and OA rat model joints tissues.

The results of H&E staining were shown in Fig. 7A. In the control group, the growth plate of the joint was well arranged, the cells were normal and healthy, and no infiltration of inflammatory immune cells was observed. In the OA group, the infiltration of inflammatory immune cells was evidently increased and the number of healthy cells decreased dramatically; as well, the arrangement of cells was chaotic. Consistently, the safranin O/fast green results revealed that the control group showed no histopathological changes with intact knee joints and richer proteoglycan in the control group, while severe joint wear and loss of proteoglycan were observed in the OA group (Fig. 7B). According to the ELISA results (Fig. 7C), compared with control group, the concentrations of TNF-α, IL-1β, and IL-6 in the serum of OA group rats were significantly increased (*p* < 0.01). As shown in the RT-qPCR results (Fig. 7D), compared with control group, the expression levels of *MS4A6A*, *C1QB*, *C1QC*, *CD74*, *CSF1R*, *HLA-DPA1*, *HLA-DRA* and *ITGB2* were all significantly increased in the joint tissues of the OA group (*p* < 0.01).

## Discussion

The pathogenic mechanisms of OA are complicated and interactive, which are manifested as articular cartilage degeneration, osteophyte formation, subchondral sclerosis, synovitis, and meniscus degeneration, respectively^20^. Therefore, it is in urgent need to expand the research and discovery of hub genes identification for therapeutic selections of OA. In this study, we analyzed GSE46750 and GSE98918 datasets related to OA using microarray data, and identified 43 co-DEGs, followed by functional enrichment analysis. Then 8 hub genes were extracted by construction of PPI network, which were further verified in vivo OA rat model.

Bioinformatics is a new reliable method combining molecular biology and information technology for predicting potential therapeutic targets of diseases, which has been conducted on OA. For instance, 161 co-DEGs are screened, of which 8 genes are finally identified as hub genes of OA synovial tissues through PPI network analysis^21^. Three microarray datasets for human synovial tissue were downloaded from GEO database, and *ATF3* has been found as a potential diagnostic marker in early diagnosis and treatment of OA^22^. Additionally, a total of 13 most overlapping DEGs in knee OA are identified from four distinct joint tissues of OA, including articular cartilage, synovial membrane, subchondral bone, and meniscus^23^. Herein, the gene expression profiles derived from synovial membrane tissues and meniscus are covered, followed by identifying overlapping DEGs and 8 hub genes including *MS4A6A*, *C1QB*, *C1QC*, *CD74*, *CSF1R*, *HLA-DPA1*, *HLA-DRA* and *ITGB2* were screened from our constructed PPI network.

Among these hub genes, colony stimulating factor-1 receptor (*CSF-1R*) plays crucial roles in innate immunity by modulating the development of most tissue macrophages and osteoclasts^24^. It has also been observed that *CSF-1R* inhibition abrogates cartilage degradation, bone erosion, and systemic bone loss in mice, which is associated with osteoclasts depletion^25^. Integrin subunit beta 2 (*ITGB2*) is highly expressed in patients with OA, and its level increased with the elevation of OA grade, which is related to the severity of OA, suggesting an independent risk factor of OA^26^. It is implicated that *ITGB2* is involved in the process of phosphate metabolic pathway given the severe meniscal calcification in OA menisci^27^. A study suggested that *ITGB2* and *HLA-DPA1* are significantly increased in OA meniscal compared with the control group^28^. *C1QB*, *C1QC* and *C1QA* are expressed in primary human articular chondrocytes and upregulated at mRNA levels with pro-inflammatory cytokines stimulation of chondrocytes^29^. Another study has reported that *C1QB*, *C1QC* and *ITGB2* identified immune-related hub genes have been recognized as diagnostic biomarkers of OA^30^. *CD74*, a type II transmembrane protein is essential for macrophage migration inhibitory factor to affect receptor activator of NF-κB downstream signaling and regulate bone mass of mice^31^. A study also has revealed that human leukocyte antigen-DR alpha (*HLA-DRA*) identified is upregulated in the chondrocytes from normal people treated with IL-1β^32^. Membrane spanning 4-domains A6A (*MS4A6A*) has also been shown to be associated with aging-related neurodegenerative diseases. High levels of *MS4A6A* in brain and peripheral blood were proposed to be positively correlate with Alzheimer's disease pathology. The specific role of MS4A6A in OA progression remains to be further investigated. In the present study, upregulation of *MS4A6A*, *C1QB*, *C1QC*, *CD74*, *CSF1R*, *HLA-DPA1*, *HLA-DRA* and *ITGB2* were observed in the joint tissues of the OA rat model, which may provide a resource for future pathological studies of OA.

Based on an in-depth bioinformatic analysis of the two datasets and GO/KEGG enrichment analysis results, we concluded that DEGs were enriched in the terms complement activation”, “clathrin-coated endocytic vesicle membrane”, MHC class II receptor activity and “complement component C3b binding. The enriched pathways were mainly “complement and coagulation cascades pathway”, “rheumatoid arthritis pathway”, and “antigen processing and presentation pathway”. Consistently, it has been found that higher synovial fluid complement factors (C5) levels are linked to elevated complement activation and reduced synovial vascularization in males with OA^33^. Our results are also in agreement with previously reported findings that inflammation, innate immunity, and activation and high expression of matrix metalloproteinases in synovitis and cartilage degeneration are associated with pathological progression of OA^34,35^.

There are several limitations of the study to consider. Firstly, although we originally intended to screen as many data sets as possible, only two expression profiles derived from synovial membrane and meniscus were covered into the data analysis. Besides, due to difficulties in obtaining appropriate clinical specimens, only OA bilateral knee joints specimens in rats were used to carry out obtained hub genes validation experiments. In addition, the relative expression of our identified hub genes in cartilage human samples or other joint components and the specific functions in OA progression are needed to be further validated.

## Conclusions

In conclusion, through a comprehensive bioinformatics analysis, we identified 8 hub genes associated with OA in synovitis tissues and meniscus tissues. These genes can be considered potential disease markers to discriminate OA patients from healthy individuals.

### Supplementary Information

Supplementary Figures.

Supplementary Tables.

## Data Availability

All data in this study are available by contacting corresponding authors.
