# Supplementary material for: Bioinformatics analysis of hub genes as osteoarthritis prognostic biomarkers
Source: Sci Rep. 2023 Dec 21;13:22894. doi: 10.1038/s41598-023-48446-1 (PMC10739719; doi:10.1038/s41598-023-48446-1)

**Supplement Figure 1**

**Bar graphs of GO and KEGG pathway enrichment analysis results.**

(A) Bar graph of the DEGs’ GO enrichment analysis results. The x-axis represents the GO terms. The y-axis represents the -log10 (p value) of each GO term. (B) Bar graph of the DEGs’ KEGG pathway enrichment analysis results.


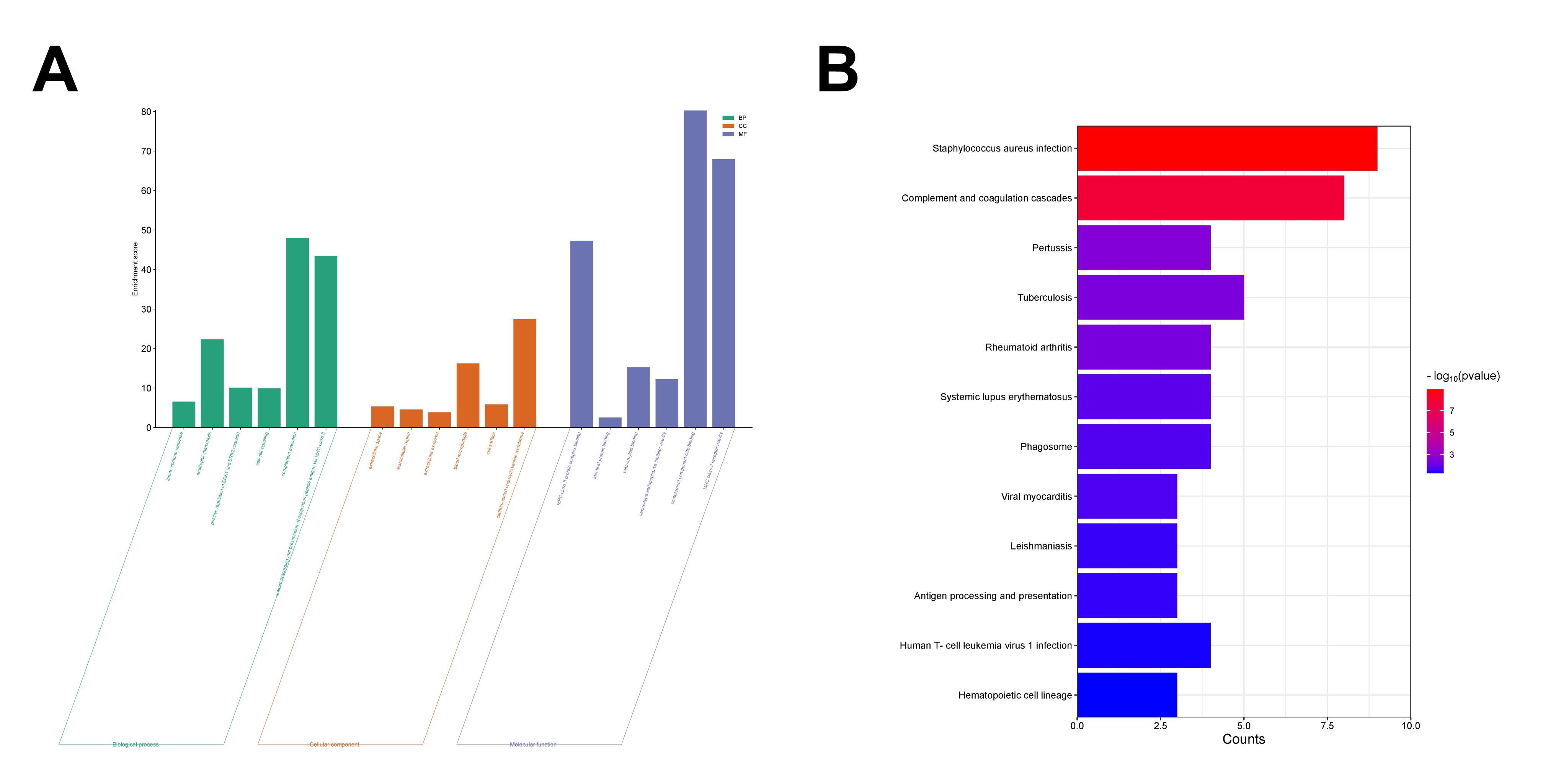


**Supplement Figure 2**

**Construction of a Protein-protein interaction (PPI) network.**

**
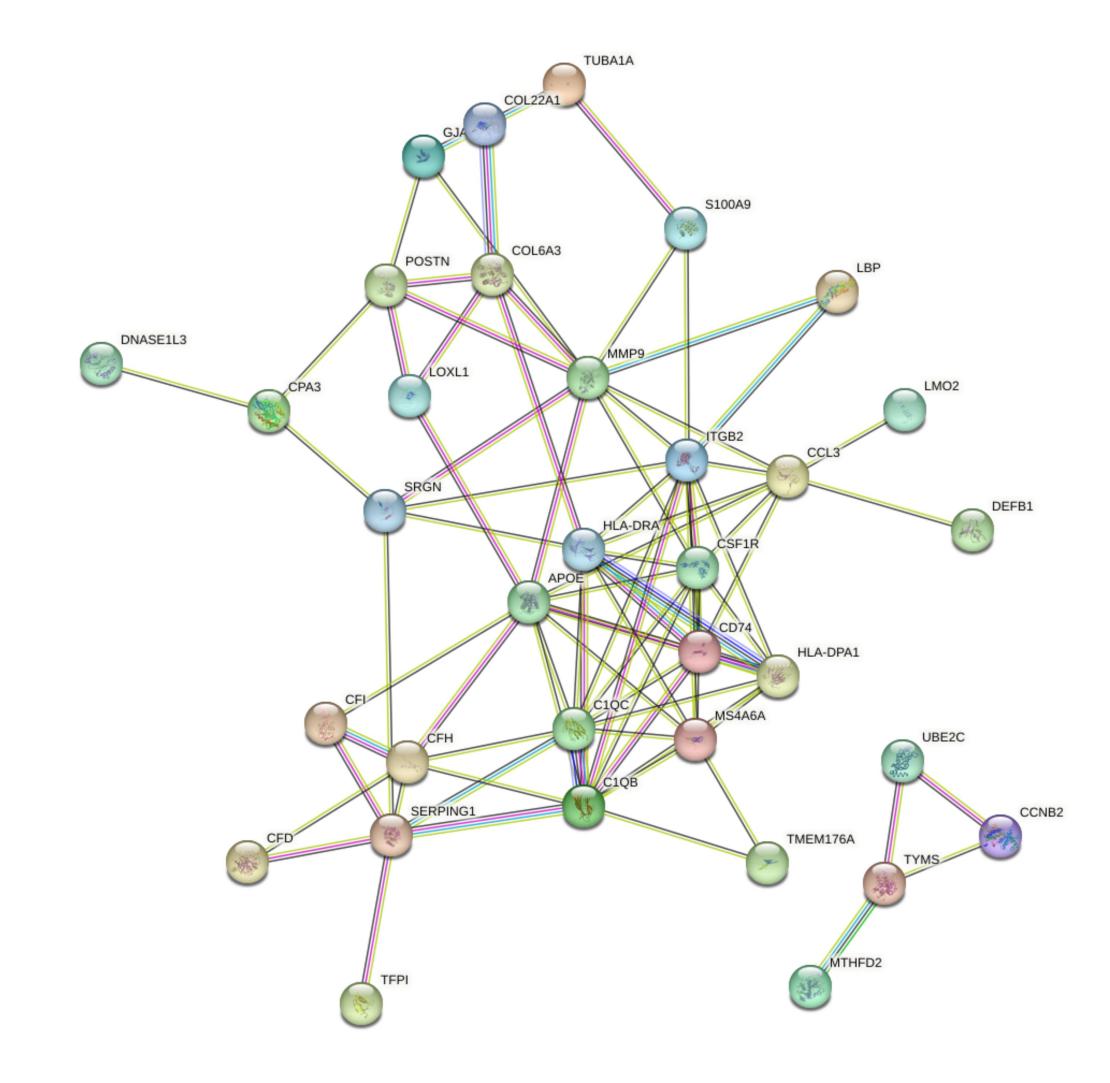
**

**Supplement Figure 3**

**The ridgeplot of hub genes.**

The x-axis represents gene expression levels. The shape of ridgeplot of each gene represents the degree of dispersion within the dataset. The height of each peak represents the corresponding sample numbers of the gene expression levels.


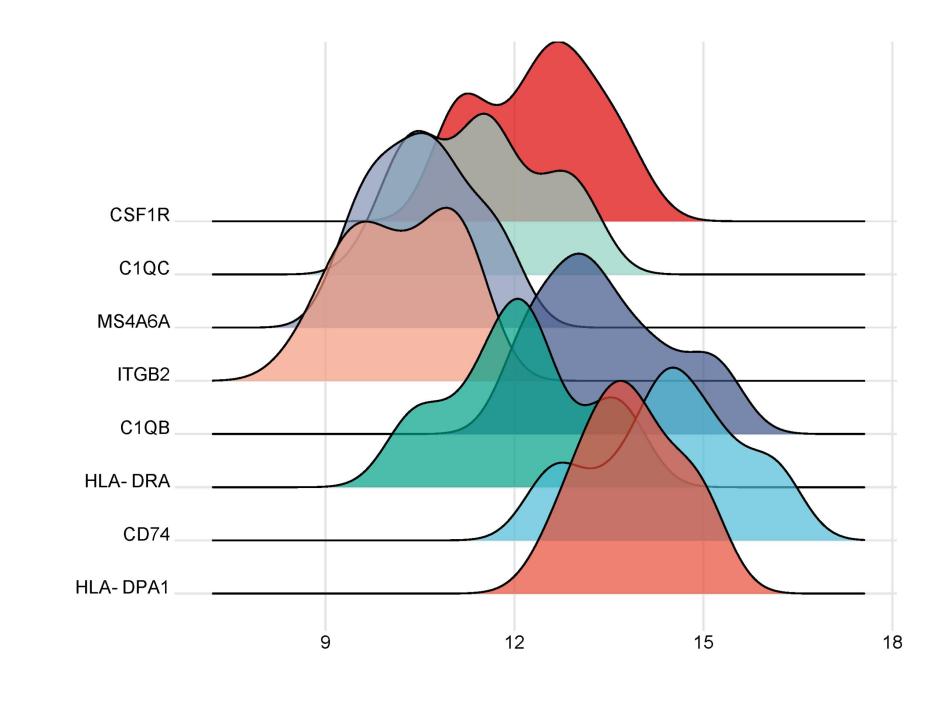

Supplement: Supplementary file 1 — Supplementary Figures. [file 41598_2023_48446_MOESM1_ESM.docx]
