# Supplementary material for: Bioinformatics analysis of hub genes as osteoarthritis prognostic biomarkers
Source: Sci Rep. 2023 Dec 21;13:22894. doi: 10.1038/s41598-023-48446-1 (PMC10739719; doi:10.1038/s41598-023-48446-1)
Supplement: Supplementary file 2 — Supplementary Tables. [file 41598_2023_48446_MOESM2_ESM.docx]

Supplementary Table 1

Primer Sequences.

| Name | Sequence（5’-3’） |
| --- | --- |
| GAPDH-F | CTCATGACCACAGTCCATGC |
| GAPDH-R | TTCAGCTCTGGGATGACCTT |
| MS4A6A-F | TCACAACCTGTTCCCAATGA |
| MS4A6A-R | AGAAGGAAGCAGATGCCAAA |
| C1QB-F | AGGAGAGAAAGGGCTTCCAG |
| C1QB-R | TTTTCTGGGTGGCCTTGTAG |
| C1QC-F | TAGACCATTCTCCCCACCAG |
| C1QC-R | TATATCTCCCCACCCCTTCC |
| CD74-F | TAGACAGATCCCCGTTCCTG |
| CD74-R | TGGAAAACATTGGCTCTTCC |
| CSF1R-F | TGGAGAGACCCACCTTCAAC |
| CSF1R-R | TCGTCCTGCATCACAGAGTC |
| HLA-DPA1-F | TCTTCCTGCCCAGAACAGAT |
| HLA-DPA1-R | GCACAGTCTCCGTTGTCTCA |
| HLA-DRA-F | TCCCTTGGTTTGTGAAGAGG |
| HLA-DRA-R | GTAGAGACGGGGTTTCACCA |
| ITGB2-F | TCTGCTTCTGCGGGAAGT |
| ITGB2-R | CTGGTAGCCTGAATGGCACT |

Supplementary Table 2

Top 20 up-regulated and down-regulated genes in GSE46750 dataset.

| Name | Description | log2FoldChange | pval | up/down |
| --- | --- | --- | --- | --- |
| SERPINE1 | serpin family E member 1 | -1.70 | 0.01 | down |
| PSAT1 | phosphoserine aminotransferase 1 | -1.68 | 0.01 | down |
| POSTN | periostin | -1.62 | 0.00 | down |
| F2R | coagulation factor II thrombin receptor | -1.49 | 0.00 | down |
| CRIP1 | cysteine rich protein 1 | -1.47 | 0.00 | down |
| PODXL | podocalyxin like | -1.44 | 0.00 | down |
| PTX3 | pentraxin 3 | -1.33 | 0.02 | down |
| PHGDH | phosphoglycerate dehydrogenase | -1.27 | 0.01 | down |
| ACAN | aggrecan | -1.26 | 0.00 | down |
| DKK1 | dickkopf WNT signaling pathway inhibitor 1 | -1.24 | 0.02 | down |
| CAP2 | CAP, adenylate cyclase-associated protein, 2 (yeast) | -1.17 | 0.02 | down |
| TK1 | thymidine kinase 1 | -1.17 | 0.01 | down |
| UBE2C | ubiquitin conjugating enzyme E2 C | -1.16 | 0.02 | down |
| KIAA0101 | KIAA0101 | -1.14 | 0.02 | down |
| SOX8 | SRY-box 8 | -1.14 | 0.00 | down |
| CEP55 | centrosomal protein 55 | -1.12 | 0.01 | down |
| FOXM1 | forkhead box M1 | -1.09 | 0.00 | down |
| CSPG4 | chondroitin sulfate proteoglycan 4 | -1.09 | 0.00 | down |
| CRIP2 | cysteine rich protein 2 | -1.09 | 0.00 | down |
| TREM1 | triggering receptor expressed on myeloid cells 1 | 1.79 | 0.00 | up |
| MMP9 | matrix metallopeptidase 9 | 1.82 | 0.00 | up |
| CPA3 | carboxypeptidase A3 | 1.84 | 0.01 | up |
| C1QC | complement C1q C chain | 1.85 | 0.00 | up |
| C11orf96 | chromosome 11 open reading frame 96 | 1.86 | 0.00 | up |
| RASD1 | ras related dexamethasone induced 1 | 1.86 | 0.00 | up |
| APOE | apolipoprotein E | 1.87 | 0.00 | up |
| PPBP | pro-platelet basic protein | 1.89 | 0.01 | up |
| CXCL6 | C-X-C motif chemokine ligand 6 | 1.97 | 0.01 | up |
| TPSAB1 | tryptase alpha/beta 1 | 1.98 | 0.01 | up |
| LYZ | lysozyme | 2.09 | 0.00 | up |
| HSD11B1 | hydroxysteroid 11-beta dehydrogenase 1 | 2.09 | 0.00 | up |
| PLA2G2A | phospholipase A2 group IIA | 2.09 | 0.01 | up |
| SPON1 | spondin 1 | 2.11 | 0.01 | up |
| CXCL5 | C-X-C motif chemokine ligand 5 | 2.13 | 0.01 | up |
| CXCL8 | C-X-C motif chemokine ligand 8 | 2.15 | 0.01 | up |
| HLA-DRA | major histocompatibility complex, class II, DR alpha | 2.17 | 0.00 | up |
| RNASE1 | ribonuclease A family member 1, pancreatic | 2.30 | 0.00 | up |
| STC1 | stanniocalcin 1 | 2.54 | 0.00 | up |
| LBP | lipopolysaccharide binding protein | 2.94 | 0.00 | up |

Supplementary Table 3

Top 20 up-regulated and down-regulated genes in GSE98918 dataset.

| Name | Description | log2FoldChange | pval | up/down |
| --- | --- | --- | --- | --- |
| XLOC_l2_004647 | XLOC_l2_004647 | -3.25 | 0.00 | down |
| MRPS27 | Mitochondrial Ribosomal Protein S27 | -2.93 | 0.00 | down |
| VEGFA | Vascular Endothelial Growth Factor A | -2.85 | 0.00 | down |
| ADAM12 | ADAM Metallopeptidase Domain 12 | -2.76 | 0.00 | down |
| POSTN | Periostin | -2.59 | 0.00 | down |
| CEMIP | Cell Migration Inducing Hyaluronidase 1 | -2.32 | 0.00 | down |
| NOL4 | Nucleolar Protein 4 | -2.30 | 0.00 | down |
| PLEKHG4 | Pleckstrin Homology And RhoGEF Domain Containing G4 | -2.19 | 0.00 | down |
| MEG9 | Maternally Expressed 9 | -2.19 | 0.00 | down |
| MFI2-AS1 | GeneCards Symbol: MELTF-AS1 | -2.18 | 0.00 | down |
| HOXB2 | Homeobox B2 | -2.14 | 0.00 | down |
| ADAMTS14 | ADAM Metallopeptidase With Thrombospondin Type 1 Motif 14 | -2.12 | 0.00 | down |
| SGK2 | Serum/Glucocorticoid Regulated Kinase 2 | -2.06 | 0.00 | down |
| COL6A3 | Collagen Type VI Alpha 3 Chain | -2.05 | 0.00 | down |
| MEG3 | Maternally Expressed 3 | -1.99 | 0.00 | down |
| PAX8-AS1 | PAX8 Antisense RNA 1 | -1.99 | 0.00 | down |
| PITPNM3 | PITPNM Family Member 3 | -1.94 | 0.01 | down |
| SPON2 | Spondin 2 | -1.93 | 0.01 | down |
| MRI1 | Methylthioribose-1-Phosphate Isomerase 1 | -1.92 | 0.00 | down |
| lnc-RTN2-1 | GeneCards Symbol: lnc-RTN2-1 | 1.82 | 0.01 | up |
| lnc-ERGIC2-1 | GeneCards Symbol: lnc-ERGIC2-1 | 1.82 | 0.06 | up |
| TSPAN7 | Tetraspanin 7 | 1.82 | 0.00 | up |
| ELTD1 | Adhesion G Protein-Coupled Receptor L | 1.87 | 0.00 | up |
| PLA2G2A | Phospholipase A2 Group IIA | 1.88 | 0.00 | up |
| VWF | Von Willebrand Facto | 1.88 | 0.00 | up |
| LPHN2 | Adhesion G Protein-Coupled Receptor L2 | 2.00 | 0.00 | up |
| HBB | Hemoglobin Subunit Beta | 2.00 | 0.10 | up |
| DEFA3 | Defensin Alpha 3 | 2.01 | 0.00 | up |
| GRM7 | Glutamate Metabotropic Receptor 7 | 2.09 | 0.06 | up |
| CFD | Complement Factor D | 2.11 | 0.00 | up |
| LOC102724332 | LOC102724332 | 2.15 | 0.01 | up |
| SPARCL1 | SPARC Like 1 | 2.19 | 0.00 | up |
| lnc-C2orf40-5 | lnc-C2orf40-5 | 2.22 | 0.01 | up |
| CD34 | CD34 Molecule | 2.29 | 0.00 | up |
| lnc-AP1S2-2 | Novel Transcript, Antisense To MAGEB17 | 2.44 | 0.00 | up |
| S100A8 | S100 Calcium Binding Protein A8 | 2.49 | 0.00 | up |
| RGS5 | Regulator Of G Protein Signaling 5 | 2.58 | 0.01 | up |
| ABCC9 | ATP Binding Cassette Subfamily C Member 9 | 2.58 | 0.00 | up |
| CSN1S1 | Casein Alpha S1 | 2.86 | 0.00 | up |

Supplementary Table 4

Top 6 GO terms of each category for DEGs between Control and OA.

| Category | Term | Genes | Count | PValue | Fold Enrichment |
| --- | --- | --- | --- | --- | --- |
| BP | GO:0045087~innate immune response | C1QB, BST2, CSF1R, CFI, SERPING1, DEFB1, LBP, S100A9, C1QC | 9 | 0.00 | 6.85 |
| BP | GO:0030593~neutrophil chemotaxis | ITGB2, CCL3, LBP, S100A9 | 4 | 0.00 | 22.45 |
| BP | GO:0006956~complement activation | C1QB, CFD, CFH | 3 | 0.00 | 55.24 |
| BP | GO:0070374~positive regulation of ERK1 and ERK2 cascade | CD74, CSF1R, PLA2G2A, CCL3, APOE | 5 | 0.00 | 10.05 |
| BP | GO:0007267~cell-cell signaling | GJA1, FGFBP2, ITGB2, CCL3, S100A9 | 5 | 0.00 | 10.01 |
| BP | GO:0006954~inflammatory response | CSF1R, GPR68, PLA2G2A, ITGB2, CCL3, S100A9 | 6 | 0.00 | 6.69 |
| CC | GO:0005615~extracellular space | CFD, CPA3, SRGN, POSTN, FGFBP2, CFH, COL22A1, CFI, PLA2G2A, DEFB1, TFPI, MMP9, LOXL1, PODXL, MTHFD2, CCL3, SERPING1, COL6A3, LBP, APOE, S100A9, C1QC | 22 | 0.00 | 5.43 |
| CC | GO:0005576~extracellular region | C1QB, CFD, CPA3, SRGN, MEGF6, CFH, COL22A1, CFI, PLA2G2A, DEFB1, TFPI, MMP9, LOXL1, CCL3, SERPING1, COL6A3, LBP, APOE, S100A9, DNASE1L3, C1QC | 21 | 0.00 | 4.72 |
| CC | GO:0070062~extracellular exosome | CFD, CLIC6, CD74, CFH, CFI, PLA2G2A, ITGB2, DEFB1, MMP9, BST2, TUBA1A, PODXL, SERPING1, COL6A3, HLA-DRA, LBP, APOE, S100A9 | 18 | 0.00 | 3.88 |
| CC | GO:0072562~blood microparticle | C1QB, CFH, SERPING1, APOE, C1QC | 5 | 0.00 | 16.50 |
| CC | GO:0009986~cell surface | BST2, CD74, CSF1R, ITGB2, HLA-DRA, LBP, TFPI, HLA-DPA1 | 8 | 0.00 | 5.96 |
| CC | GO:0030669~clathrin-coated endocytic vesicle membrane | CD74, HLA-DRA, APOE, HLA-DPA1 | 4 | 0.00 | 27.35 |
| MF | GO:0023026~MHC class II protein complex binding | CD74, HLA-DRA, HLA-DPA1 | 3 | 0.00 | 48.86 |
| MF | GO:0042802~identical protein binding | C1QB, BST2, CD74, TUBA1A, CFH, LMO2, CCL3, DEFB1, APOE, MMP9 | 10 | 0.01 | 2.55 |
| MF | GO:0001540~beta-amyloid binding | CD74, ITGB2, APOE | 3 | 0.02 | 15.34 |
| MF | GO:0004867~serine-type endopeptidase inhibitor activity | SERPING1, COL6A3, TFPI | 3 | 0.02 | 12.81 |
| MF | GO:0001851~complement component C3b binding | CFH, ITGB2 | 2 | 0.02 | 79.95 |
| MF | GO:0032395~MHC class II receptor activity | HLA-DRA, HLA-DPA1 | 2 | 0.03 | 67.65 |

Supplementary Table 5

Top 10 KEGG pathways for DEGs between Control and OA.

| Category | Term | Genes | Count | PValue | Fold Enrichment |
| --- | --- | --- | --- | --- | --- |
| KEGG_PATHWAY | hsa05150:Staphylococcus aureus infection | C1QB, CFD, CFH, CFI, ITGB2, HLA-DRA, DEFB1, HLA-DPA1, C1QC | 9 | 0.00 | 24.04 |
| KEGG_PATHWAY | hsa04610:Complement and coagulation cascades | C1QB, CFD, CFH, CFI, ITGB2, SERPING1, TFPI, C1QC | 8 | 0.00 | 23.85 |
| KEGG_PATHWAY | hsa05133:Pertussis | C1QB, ITGB2, SERPING1, C1QC | 4 | 0.00 | 13.50 |
| KEGG_PATHWAY | hsa05152:Tuberculosis | CD74, ITGB2, HLA-DRA, LBP, HLA-DPA1 | 5 | 0.00 | 7.12 |
| KEGG_PATHWAY | hsa05323:Rheumatoid arthritis | ITGB2, CCL3, HLA-DRA, HLA-DPA1 | 4 | 0.01 | 11.03 |
| KEGG_PATHWAY | hsa05322:Systemic lupus erythematosus | C1QB, HLA-DRA, HLA-DPA1, C1QC | 4 | 0.01 | 7.54 |
| KEGG_PATHWAY | hsa04145:Phagosome | TUBA1A, ITGB2, HLA-DRA, HLA-DPA1 | 4 | 0.02 | 6.75 |
| KEGG_PATHWAY | hsa05416:Viral myocarditis | ITGB2, HLA-DRA, HLA-DPA1 | 3 | 0.02 | 12.82 |
| KEGG_PATHWAY | hsa05140:Leishmaniasis | ITGB2, HLA-DRA, HLA-DPA1 | 3 | 0.03 | 9.99 |
| KEGG_PATHWAY | hsa04612:Antigen processing and presentation | CD74, HLA-DRA, HLA-DPA1 | 3 | 0.03 | 9.86 |
